# The complete chloroplast genome of *Mimosa pudica* and the phylogenetic analysis of mimosoid species

**DOI:** 10.1080/23802359.2018.1532831

**Published:** 2018-10-26

**Authors:** Xiaoyue Yang, Xiyou Qian, Zefu Wang

**Affiliations:** a State Key Laboratory of Grassland Agro-Ecosystem, School of Life Sciences, Lanzhou University, Lanzhou, People’s Republic of China;; b Heilongjiang Institute of Forestry Monitoring and Planning, Harbin, People’s Republic of China;; c Key Laboratory of Bio-Resource and Eco-Environment of Ministry of Education, College of Life Sciences, Sichuan University, Chengdu, People’s Republic of China

**Keywords:** Complete chloroplast genome, *Mimosa pudica*, phylogenetic analysis, mimosoid species

## Abstract

*Mimosa pudica*, a typical sensitive plant, belongs to *Mimosa* (Fabaceae). It is well known for the rapid plant movement. In this study, we determined the complete chloroplast genome of *M. pudica* using the Illumina reads. The complete chloroplast genome of *M. pudica* is 163,237 bp in length, comprising a large single-copy region (LSC) of 92,045 bp, a small single-copy region (SSC) of 18,786 bp, and a pair of inverted repeats (IRs) of 26,203 bp each. The genome contained 129 encoded genes in total, including 84 protein-coding genes, eight ribosomal RNA genes, and 37 transfer RNA genes. The overall GC content of the *M. pudica* chloroplast genome is 35.53%. We reconstructed the phylogenetic relationships for 19 mimosoid species. It was revealed that *M. pudica* was closely related to *Piptadenia communis*.

*Mimosa pudica* (*Mimosa*, Fabaceae) is a typical sensitive plant. It is well known for the rapid plant movement and often grown for people’s curiosity. A recent study has reported the *de novo* genome of *M. pudica* (Griesmann et al. 2018); however, its complete chloroplast genome has not been analyzed or provided for further study. Herein, we assembled and annotated the complete chloroplast genome of *M. pudica*, and performed a phylogenetic analysis including 19 species of mimosoid clade.

We downloaded ∼20G high-quality Illumina reads of *M. pudica* (NCBI accession # SRR5313988) using the fastq-dump software. The individual for sequencing was collected in DC Smith greenhouse, University of Wisconsin, MI, and the sample was stored at Toulouse Henry Gaussen Botanical Garden in France with the accession number FR-0-TOU-D18001N (Griesmann et al. 2018). The reads were used to assemble the complete chloroplast genome with the software NOVOPlasty v2.5.9 (Dierckxsens et al. 2017) and GapCloser (Luo et al. 2012). We performed the annotation with the software Plann (Huang and Cronk 2015) and Sequin (NCBI website). Finally, we obtained a chloroplast genome of *M. pudica*. The genome has been submitted to GenBank under the accession number of MH671330.

The complete chloroplast genome of *M. pudica* is 163,237 bp in length, containing a large single-copy (LSC) region with of 92,045 bp, a small single-copy (SSC) region of 18,786 bp, and a pair of inverted repeats (IRA and IRB) with a length of 26,203 bp each. The GC content of *M. pudica* chloroplast genome is 35.53% in total. The SSC region has lower GC content (29.84%) than the LSC (32.68%) and IR (42.59%) regions. The chloroplast genome of *M. pudica* contains 129 genes, including 84 protein-coding genes (PCG), eight ribosomal RNA (rRNA) genes, and 37 transfer RNA (tRNA) genes. Most genes are single copy; however, 17 genes (six PCGs, four rRNA genes, and seven tRNA genes) are duplicated in the IR regions.

To infer the phylogenetic position of *M. pudica* and estimate the phylogenetic relationships of mimosoid clade, we reconstructed a phylogenetic tree including 19 mimosoid species (including *M. pudica*) based on their complete chloroplast genomes. *Senna occidentalis* (*Senna*, Fabaceae), a species of cassia clade, was used as an outgroup. First, we extracted the coding sequences (CDS) from 76 protein-coding genes (PCGs), which were shared by all of 20 species. For each gene, PRANK (Löytynoja and Goldman 2008) was used to align the sequences. Then, each pair of the aligned sequences was concatenated into a supermatrix. The software RAxML (Stamatakis 2014) was performed to construct the phylogenetic tree with 100 bootstrap replicates. The ML tree revealed *M. pudica* was closely related to *Piptadenia communis* (*Piptadenia*, Fabaceae) with strongly support (Figure 1).

**Figure 1.**
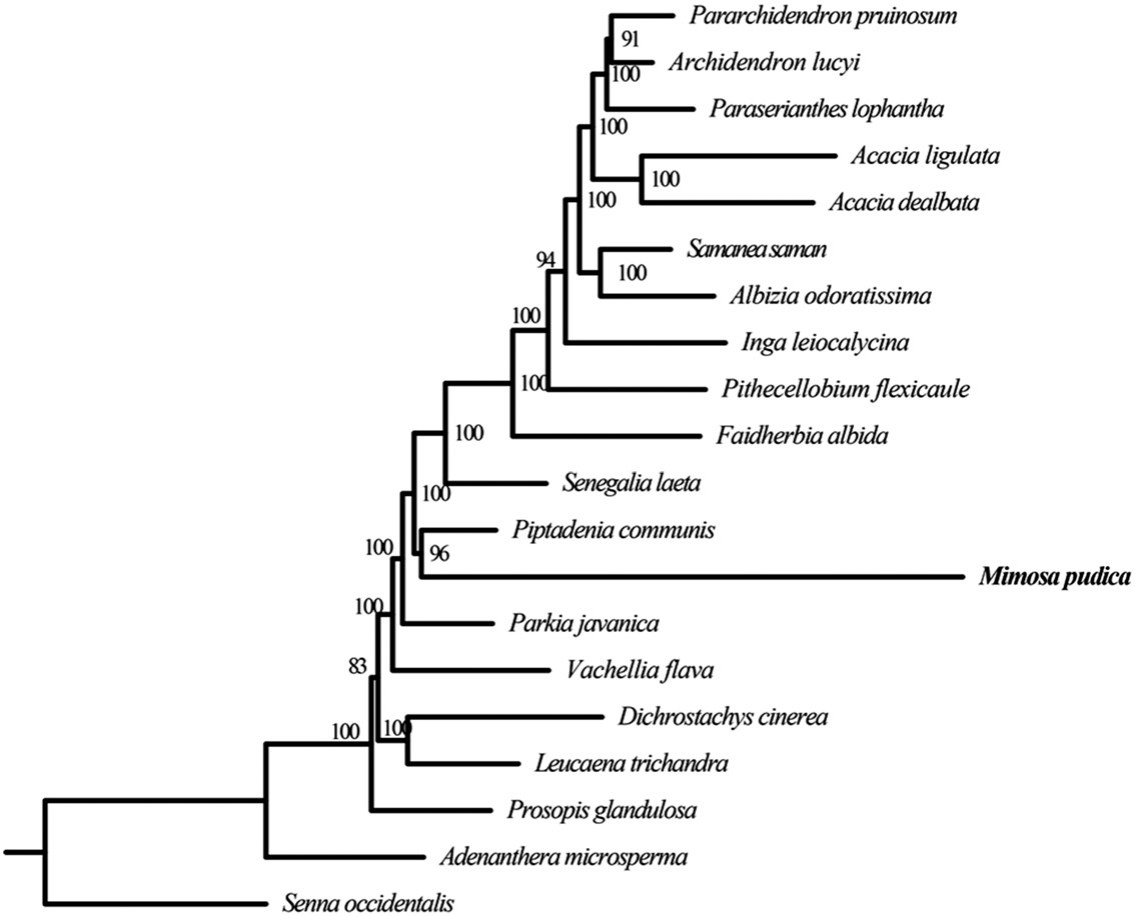
Maximum-likelihood (ML) tree based on the concatenated data of 76 PCGs sequences of *Mimosa pudica* and 19 other species. Numbers in the nodes are the bootstrap values. Their accession numbers are as follows: *Pararchidendron pruinosum*: NC_035348.1, *Archidendron lucyi*: NC_034988.1, *Paraserianthes lophantha*: LN885334.1, *Acacia ligulata*: NC_026134.1, *Acacia dealbata*: NC_034985.1, *Samanea saman*: NC_034992.1, *Albizia odoratissima*: NC_034987.1, *Inga leiocalycina*: NC_028732.1, *Pithecellobium flexicaule*: NC_034991.1, *Faidherbia albida*: NC_035347.1, *Senegalia laeta*: NC_036736.1, *Piptadenia communis*: NC_034990.1, *Parkia javanica*: NC_034989.1, *Vachellia flava*: NC_036734.1, *Dichrostachys cinerea*: NC_035346.1, *Leucaena trichandra*: NC_028733.1, *Prosopis glandulosa*: KJ468101, *Adenanthera microsperma*: NC_034986.1, *Senna occidentalis*: MF358692.1.

Above all, we provide a valuable genomic information of *M. pudica*. The phylogenetic tree we reconstructed could yield a better understanding for us about the mimosoid species.
